# MRI for managing intermediate & low risk prostate cancer

**DOI:** 10.1186/1470-7330-14-S1-O9

**Published:** 2014-10-09

**Authors:** Anwar R Padhani

**Affiliations:** 1 Mount Vernon Cancer Centre, London, UK

## Intermediate risk disease

Pathological status: PSA 10–20 ng/mL, **or** biopsy Gleason score 7, **or** clinical stage T2b or T2c

Clinical note: Heterogeneous group with a wide incidence of biochemical relapse & numerous curative therapy options.

Problems with categorization: Detection of unfavourable subgroup includes Gleason ≥4+3 *and/or* >50% positive biopsies *and/or* >1 intermediate risk factors [1,2]

Role of MRI in practice:

1. If initial active surveillance is considered, then it is important not to underestimate tumor grade/ volume/stage

2. For external beam radiotherapy, the presence of unfavourable disease affects duration of adjuvant hormonal therapy.

3. For focal therapy, index lesion localization is needed

4. For surgery, accurate staging to enable curative treatment with negative margins & nerve sparing if possible

Type of MRI [3]:

• Lesion detection and localisation protocol with T2W, DW-MRI and DCE-MRI ± MRSI for low ADC lesions to assess aggressiveness

• Staging with multi-planar T2W, DW-MRI ± DCE-MRI for ECE/SVI

## High risk disease

Pathological status: PSA >20 ng/mL, **or** Gleason score 8–10, **or** clinical stage >T2c

Clinical note: Highest risk of biochemical recurrence and cancer specific mortality but substantial population heterogeneity

Prognostic subgroups [4]:

■ Good prognosis subgroup: one single risk factor (any)

■ Intermediate prognosis subgroup: two risk factors (PSA >20 ng/ml and stage cT3–4); **No** Gleason 8+ disease

■ Poor prognosis subgroup: GS >7 and stage cT3–4 and/or PSA >20 ng/ml

Clinical sub-groups: localized, locally advanced & metastatic

Problems with categorization: Local staging accuracy & the detection of metastatic disease

Role of MRI in practice

1. local and nodal staging: to detect extensive ECE/SVI that would preclude radical surgery with negative margins. Nerve sparing rarely undertaken.

2. To detect nodal and bone metastases

Type of MRI [3,5]:

• Accurate local staging and pelvic nodal assessments

• Bone scan + CT abdomen or WB-MRI
